# Exposure Assessment of the Tehran Population (Iran) to Zearalenone Mycotoxin

**Published:** 2012

**Authors:** Hassan Yazdanpanah, Afshin Zarghi, Ali Reza Shafaati, Seyyed Mohsen Foroutan, Farshid Aboul-Fathi, Arash Khoddam, Firoozeh Nazari

**Affiliations:** a *School of Pharmacy, Shahid Beheshti University of Medical Sciences, Tehran, Iran.*; b *Food and Drug Deputy, Iranian Ministry of Health and Medical Education, Tehran, Iran.*; c *Noor Research and Educational Institute, Tehran, Iran.*; d *Student Research committee, School of Pharmacy, Shahid Beheshti University of Medical Sciences, Tehran, Iran. *

## Abstract

Zearalenone (ZEA) mycotoxin is a potent estrogenic metabolite. It is the primary toxin causing infertility, abortion or other breeding problems. A HPLC method was validated for ZEA in foods using a monolithic column with sample clean-up on an immunoaffinity column. A certified reference material (CRM) from FAPAS (UK) was analyzed. A survey of ZEA was performed on the 72 samples of rice, bread, puffed corn snack and wheat flour collected from Tehran retail market. The average recovery and coefficient of variation in different foods ranged 92.7-107.1 and 4.9-13.8%, respectively. The amount of ZEA in corn CRM was in the acceptable range of FAPAS. The limit of quantification was 3 ng/g for rice, bread and wheat flour and 2.7 ng/g for puffed corn snack. The retention time of zearalenone was 2.6 min. All samples had contamination level lower than the maximum tolerated level of ZEA in foods in Iran. The mean intake of ZEA from all samples was much lower than the tolerable daily intake estimated by JECFA. This is the first survey on ZEA contamination in bread and rice in Iran as well as the first study on exposure assessment of Tehran population to ZEA.

## Introduction

Zearalenone (ZEA) is an estrogenic compound produced by *Fusarium spp*. such as *F. graminearum *and *F. culmorum *(1). It is associated with reproductive problems in specific animals and possibly in humans (2). ZEA may affect the uterus by decreasing progesterone secretion and altering the morphology of uterine tissues (3). It is classified by the international agency for research on cancer under group 3 carcinogens (4). 

ZEN is found worldwide in a number of cereal crops, such as maize, barley, wheat, rice (5, 6), and bread (7). It is generally stable during cooking, except under alkaline conditions or during extrusion (8). Because of ZEA potential health hazards to humans, many countries have recently been documented regulatory levels for ZEA in the range of 20 to 1000 ng/g in foods (9). Iran has made the regulatory limits 200 ng/g for wheat, rice, and maize and 400 ng/g for barley (10). The Joint FAO/WHO Expert Committee on Food Additives (JECFA) established a provisional maximum tolerable daily intake (PMTDI) of 0.5 μg/kg body weight (11). 

Mycotoxin contaminations of foodstuffs and feedstuffs have been studied in Iran (12-16). There are little data on the natural occurrence of ZEA in cereals and cereal products in Iran. ZEA was found in wheat (17), maize (18), corn flour (19), corn cheese snack (19), barley, corn, silage and wheat bran (20). The aims of this study were determination of ZEA in different food samples collected from Tehran retail markets and to estimation of ZEA intake by the Tehran population.

## Experimental

All reagents were of analytical grade. Solvents used for the experiments were of either HPLC or analytical grade. The standard of ZEA was purchased from Sigma-Aldrich. The IAC EASI-EXTRACT® ZEARALENONE immunoaffinity column for ZEA was purchased from R-Biopharm Company, UK. The chromatographic apparatus consisted of a model Wellchrom K-1001 pump, a model Rheodyne 7125 injector and a model RF10AXL fluorescence detector connected to a model Eurochrom 2000 integrator, all from Knauer (Berlin, Germany). The separation was performed on Chromolith Performance (RP-18e, 100 × 4.6 mm) column from Merck (Darmstadt, Germany).

*Sampling and sample preparation*

Samples (rice, bread, wheat flour and puffed corn snack) were collected from various sales outlets in nine geographic zones in Tehran, Iran, according to the sampling plan for official control of mycotoxins in food (21). All samples were finely grounded by mill and subsamples stored in freezer at -32ºc until analysis.

*Preparation of ZEA standard*

Stock, intermediate and working standard solutions of ZEA were prepared in acetonitrile. After preparation of standard solution of ZEA (10 μg/mL), the concentration was determined using UV spectrophotometer in 274 nm. This standard was used to prepare spiking solutions and working standards of ZEA for HPLC analysis.

*Method*

The method used for ZEA extraction from samples and the chromatographic conditions were based on the European Standard, CEN/TC 275 with some modifications (22). Twenty grams of samples including rice, bread, and wheat flour samples were extracted for 1 h with acetonitrile 90% (100 mL). Puffed corn snack samples (20 g) were extracted with 100 mL acetonitrile 84% for 1 h. After filtering, 15 mL of the filtrate was diluted with 85 phosphate buffer saline (PBS) and filtered through a glass microfiber filter. After conditioning the column with 20 mL PBS, 50 mL diluted and filtered extracted was passed through immunoaffinity column at a flow rate of about 2-3 mL/min. Finally, the column was rinsed with 20 mL deionized water at the same flow rate and dried with a gentle vacuum. For ZEA elution from the column, a portion of 1.5 mL HPLC grade acetonitrile was passed through the column and then diluted with 3 mL deionized water. Finally, 100 μL of the final solution was injected into HPLC.

For all samples, separation was performed on a monolithic column (100 × 4.6 mm) using a HPLC system equipped with a fluorescence detector. Mobile phase was acetonitrile-water (55:45) with a flow rate of 1.5 mL/min. The fluorescence detector was operated at excitation wavelength of 275 nm and emission wavelength of 450 nm.

*Method validation*

To evaluate the reliability of the results, in addition to apply regular validation assessment to the developed method, internal quality control experiments were also performed. Recovery experiments were performed for determination of accuracy and precision of the method using blank rice, bread, puffed corn snack and wheat flour samples spiked at three ZEA levels (100, 200, 400 ng/g). In each working day, a blank and a spiked sample were also analyzed. According to the recovery values, ZEA levels were corrected for recoveries. In addition, a certified reference material (CRM) from FAPAS (UK) was also analyzed.

## Result and Discussion

*Method validation*

**Figure1:**
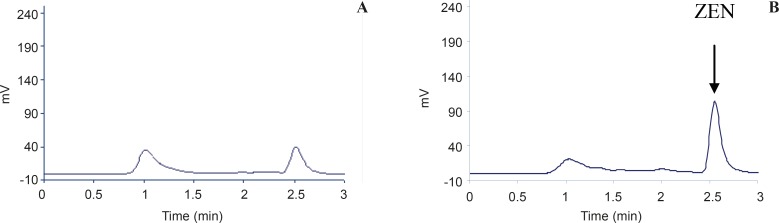
HPLC chromatograms of a: ZEA in corn CRM (313 ng/g) and b: ZEN standard (100 ng/mL).

The method was validated in terms of linearity, limit of detection (LOD, limit of quantification (LOQ), selectivity, precision and accuracy. The method was satisfactory in terms of selectivity as an IAC was applied for purification of ZEA which eliminated false positive results caused by interfering materials. Typical chromatograms obtained for ZEA are shown in Figure 1. The retention time of ZEA was very short (2.6 min).

Calibration curves were constructed using seven standards at the range of 14-1100 ng/g for puffed corn snack and 15 to 1200 ng/g for the other samples, with r^2^>0.997. Recovery experiments were performed for determination of accuracy and precision of the method using blank rice, bread, puffed corn snack and wheat flour samples spiked at three ZEA levels (100, 200, 400 ng/g). Results of the mean recovery and coefficient of variation (triplicate measurements) on all foods are shown in Table 1. 

**Table 1:** Accuracy and precision of zearalenone analytical method in different foods (n = 3).

**Sample**	**Spiking level (ng/g)**	**Recovery (%)**	**RSD**_r _ **(%) **
**Ric**e	100	93.4	17.8
	200	95.6	14.5
	400	106.1	9.1
	Mean recovery ± SD	98.4 ± 6.8	13.8
**Bread**	100	97.8	8.1
	200	116	6.1
	400	107.5	5.8
	Mean recovery ± SD	107.1 ± 9.1	6.7
**Puffed corn snack**	100	104	7.6
	200	106.8	4.7
	400	109	2.4
	Mean recovery ± SD	106.6 ± 2.5	4.9
**Wheat flour**	100	95	12.1
	200	96.3	5.3
	400	87	8.6
	Mean recovery ± SD	92.8 ± 5.0	8.7

The mean recovery and coefficient of variation in different foods ranged 92.7-107.1% and 4.9-13.8%, respectively (Table 1). These values fall well within EU method performance criteria for ZEA analysis (21). The concentration of ZEA in corn CRM was 379.4 ng/g, which was in the acceptable range of FAPAS Scheme (170-385 ng/g), confirming the accuracy of the analytical method. LOQ, signal-to-noise ratio (s/n) of 9:1 and LOD s/n 3:1 in rice, bread and wheat flour samples were 3 ng/g and 1 ng/g, respectively. For puffed corn snack, LOQ and LOD were 2.7 ng/g and 0.9 ng/g, respectively.

*Occurrence of ZEA in various foods*

This is the first study on the occurrence of ZEA in rice and bread in Iran. No ZEA contamination was observed in any of these samples (Table 2).

**Table 2:** Natural occurrence of ZEA in rice, bread, puffed corn snack and wheat flour samples marketed in Tehran, Iran

**Samples**	**Numbers of samples in the range (ng/g)**		
	< **LOQ**^a, b^	**3-200**	**≥ 200**
Rice	18	-	-
Bread	18	-	-
Wheat flour	18	-	-
Puffed corn snack^c^	18	-	-

^a^LOQ for Puffed corn snack: 2.7 ng/g. ^b^LOQ for Rice, Bread, and Wheat flour: 3 ng/g

There is little data on ZEA contamination in wheat in Iran. Karimi-Osboo reported that 15 out of 175 wheat samples (8.6%) from Golestan Province were positive for ZEA at a mean level of 72 ng/g. The range of contamination was 39-104 ng/g (17). In our study, the level of ZEA in all wheat flour samples was lower than ZEA MTL in Iran (Table 2) and was in agreement with Rashedi’s report in which no ZEA was detected in 14 wheat bran samples (20).

There is little data on ZEA contamination in corn in Iran. Hadiani et al. analysed forty preharvested maize samples from the Mazandaran province, and found 3 of 40 (7.5%) samples contained ZEA in the range 100 –212 ng/g with a mean of 141 ng/g (18). Oveisi determined ZEA in 38 corn flour and cheese snack samples. The mean of contamination was 377 ng/g and 832 ng/g in corn flour and cheese snack samples, respectively (19). Rashedi reported that 25% of corn samples were contaminated to ZEA (20). Nuryono *et al*.(2005) determined ZEA in Indonesian maize-based food and feed samples. Twenty-five out of 89 samples were contaminated in a range from 6.9 to 589 ng /g (23). In Spain, the incidence of ZEA in 25 corn-based food samples was 44 % and the level ranged from 34-216 ng/g (24). In our survey, ZEA was not found in any of puffed corn snack samples (Table 2).

*Estimation of dietary intake of ZEA*

This is the first study on exposure assessment of Tehran population to ZEA. Exposure to mycotoxins for each type of food depends on the mycotoxins concentration in food and the amount of food consumed. In this study, the consumption rates of rice and bread were based on a consumption survey performed in Iran since 2001-2003 (25). Average consumptions of rice and bread are 107 g and 286 g per day per person, respectively. Average consumption of wheat flour is 416 g per day per person (26). There is no data of puffed corn snack consumption in Iran so we assumed a package (65 g) per day as mean daily consumption. As all samples had ZEA contamination below LOQ, the ZEN level of non-contaminated samples were replaced by half the LOQ. The average body weight for adults was assumed 70 kg. In our study, estimated daily intakes of ZEA from rice, bread, wheat flour and puffed corn snack consumption was 0.002, 0.006, 0.003 and 0.001 μg/kg bw/day, respectively (Table 3). 

**Table 3:** Estimated daily intake of ZEA (μg/kg bw/day) from rice, bread, wheat flour and puffed corn snack consumption in Tehran population, Iran

**Samples**	**Mean ** ^a^ **(ng/g)**	**Daily intake (μg/kg bw/day)** ^b^
Rice	1.50	0.002
Bread	1.50	0.006
Wheat flour	1.50	0.003
Puffed corn snack^c^	1.35	0.001

a: Mean of all samples; ZEN level of non-contaminated samples were replaced by half the LOQ. b: Body weight (bw) for adults is assumed 70 kg . c: Mean weight of 65 g is assumed for each package

The total intake of ZEA from all samples consumption was 0.012 μg/kg bw/day and it was much lower than the PMTDI estimated by JECFA (0.5 μg/kg bw/day), indicating there is no health risk for consumers at these levels of contamination. In Taiwan, ZEA was detected in four out of 26 samples ranging from 7.9 to 9.0 ng/g (27). The dietary intakes of ZEA for male and female adults were 0.00297 and 0.00478 μg/kg bw/day, respectively. The ZEA dietary intakes for the Swiss population was estimated to be < 0.02 μg/kg bw/day (28). The mean dietary exposure in French population was estimated at 33 ng/kg bw/day and the 95^th ^percentile dietary exposure at 70 ng/kg bw/day (29). In 2003, the Scientific Cooperation on Questions Relating to Food (SCOOP) estimated the dietary exposure to ZEA in the European population. The mean daily dietary exposure in adult was estimated 4 to 29 ng/kg bw/day (30). Our results were in agreement with abovementioned reports in which estimated daily intakes of ZEA was much lower than the PMTDI estimated by JECFA (0.5 μg/kg bw/day) (Table 3).

## Conclusion

Although in this survey, no ZEA contamination was observed in any food samples, considering the high consumption and/or importance of these foods in Iran, more surveys are recommended. In this study which is the first one on exposure assessment of Tehran population to ZEA, no health risk for consumers were detected at these levels of contamination.

## References

[1] Yazar S, Omurtag G Z (2008). Fumonisins, trichothecenes and zearalenone in cereals. Int. J. Mol. Sci.

[2] Wood GE (1992). Mycotoxins in foods and feeds in the United States. J. Anim. Sci.

[3] Etienne M, Dourmad J Y (1994). Effects of zearalenone or glucosinolates in the diet on reproduction in sows: a review. Livest. Prod. Sci.

[4] IARC (1993). IARC Monographs on the Evaluation of Carcinogenic Risks to Humans: Some Naturally Occurring Substances: Food Items and Constituents,Heterocyclic Aromatic Amines and Mycotoxins.

[5] Kuiper-Goodman T, Scott PM, Watanabe H (1987). Risk assessment of the mycotoxin zearalenone. Reg. Toxicol. Pharmacol.

[6] Tanaka T, Hasegawa A, Yamamoto S, Lee US, Sugiura, Y, Ueno Y (1988). Worldwide contamination of cereals by the Fusarium mycotoxins, nivalenol, deoxynivalenol, and zearalenone: survey of 19 countries. J. Agric. Food Chem.

[7] Aziz NH, Attia ES, Farag SA (1997). Effect of gamma-irradiation on the natural occurrence of Fusarium mycotoxins in wheat, flour and bread. Nahrung.

[8] European Food Safety Authority (2011). Scientific opinion on the risks for public health related to the presence of zearalenone in food, EFSA panel on contaminants in the food chain (EFSA), Parma, Italy. EFSA J.

[9] (2004). Food and Agriculture Organization of the United Nations. Worldwide Regulations for Mycotoxins in Food and Feed in 2003.

[10] ISIRI (Institute of Standard and Industrial Research of Iran) (2002). Maximum Tolerated Limits of Mycotoxins in Foods and Feeds. National Standard No. 5925.

[11] JECFA (2000). Zearalenone. Joint FAO/WHO expert Committee on Food Additives.Safety Evaluation of Certain Food Additives and Contaminants.

[12] Yazdanpanah H (2006). Mycotoxin contamination of foodstuffs and feedstuffs in Iran. Iranian J. Pharm. Res.

[13] Yazdanpanah H, Eskandari Gheidari P, Zarghi A, Mirkarimi SK (2005). Survey of fumonisin B1 contamination of corn in Northern Iran during 2000. Iranian J. Pharm. Res.

[14] Ghiasian SA, Shephard GS, Yazdanpanh H (2011). Natural occurrence of aflatoxins from maize in Iran. Mycopathol.

[15] Shephard GS, Marasas WFO, Yazdanpanah H, Rahimian H, Safavi N, Zarghi A, Shafaati AR, Rasekh HR (2002). Fumonisin B1 in maize harvested in Iran during 1999. Food Addit. Contam.

[16] Fakoor Janati SS, Beheshti HR, Khoshbakht Fahim N, Feizy J (2011). Aflatoxins and ochratoxin A in bean from Iran. Bull. Environ. Contam. Toxicol.

[17] Karami-Osboo Rand Mirabolfathy M (2008). Natural zearalenone contamination of wheat from golestan province, northern Iran. Iran. J. Plant Path.

[18] Hadiani MR, Yazdanpanah H, Ghazi Khansari M, Cheraghali AM, Goodarzi M (2003). Survey of the natural occurrence of zearalenone in maize from northern Iran by thin-layer chromatography densitometry. Food Addit. Contam.

[19] Oveisi M, Hajimahmoodi M, Memarian S, Sadeghi N, Shoeibi S (2005). Determination of zearalenone in corn flour and a cheese snack product using high performance liquid chromatography with fluorescence detection. Food Addit. Contam.

[20] Rashedi M, Sohrabi HR, Ashjaazadeh MA, Azizi H, Rahimi E (2011). Zearalenone contamination in barley, corn, silage and wheat bran. Toxicol. Ind. Health.

[21] Commission regulation (EC) No (2006). 401/2006. Laying down the methods of sampling and analysis for the official control of the levels of mycotoxins in foodstuffs. Off. J. EU.

[22] MacDonald SJ, Anderson S, Brereton P, Wood R, Damant A (2005). Determination of zearalenone in barley, maize and wheat flour, polenta, and maize-based baby food by immunoaffinity column cleanup with liquid chromatography: interlaboratory study. J AOAC Int.

[23] Nuryono N, Noviandi CT, Böhm J, Razzazi-Fazeli E (2005). A limited survey of zearalenone in Indonesian maize-based food and feed by ELISA and high performance liquid chromatography. Food Control.

[24] Cerveró MC, Castillo MA, Montes R, Hernández E (2007). Determinación de tricotecenos, zearalenona y zearalenoles en alimentos derivados de maíz del mercado español. Revista Iberoamericana de Micol.

[25] National Nutrition, Food Technology Research Institute (2004). Comprehensive Study of Food Basket Pattern, and Nutrition Status in Iran during 2000-2002.

[26] Lyddon C (2010). Focus on Iran Government plan to move away from grain subsidies may have significant impact on milling industry. world-grain.com. http://www.worldgrain.com/Departments/Country%20Focus/Country%20Focus%20Home/Focus%20on%20Iran.aspx?p=1.

[27] Liao CD, Chiueh LC, Shih DYC (2009). Determination of zearalenone in cereals by high performance liquid chromatography and liquid chromatography – electrospray tandem mass spectrometry. J. Food Drug Anal.

[28] Rhyn P, Zoller O (2003). Zearalenone in cereals for human nutrition: relevant data for the Swiss population. Eur. Food Res. Tech.

[29] Leblanc JC, Tard A, Volatier JL, Verger P (2005). Estimated dietary exposure to principal food mycotoxins from the first French Total Diet Study. Food Addit. Contam.

[30] Scientific Cooperation on Questions Relating to Food (SCOOP) (2003). Task 3.2.10. Collection of Occurrence Data of Fusarium Toxins in Food and Assessment of Dietary Intake by the Population of EU Member States. Subtask II: zearalenone. EU Comm., Direct.- Gen. Health Consum. Prot.

